# The Effects of Caffeinated “Energy Shots” on Time Trial Performance

**DOI:** 10.3390/nu5062062

**Published:** 2013-06-06

**Authors:** Matthew Mark Schubert, Todd Anthony Astorino, John Leal Azevedo

**Affiliations:** 1 Department of Kinesiology, California State University Chico, 400 W. 1st Street, Yolo Hall 243, Chico, CA 95929, USA; E-Mail: jlazevedo@csuchico.edu; 2 Department of Kinesiology, California State University San Marcos, 333 S. Twin Oaks Valley Road, University Hall 320, San Marcos, CA 92096, USA; E-Mail: astorino@csusm.edu

**Keywords:** time trial, caffeine, taurine, *yerba maté*, distance running

## Abstract

An emerging trend in sports nutrition is the consumption of energy drinks and “energy shots”. Energy shots may prove to be a viable pre-competition supplement for runners. Six male runners (mean ± SD age and VO_2_max: 22.5 ± 1.8 years and 69.1 ± 5.7 mL·kg^−1^·min^−1^) completed three trials [placebo (PLA; 0 mg caffeine), Guayakí Yerba Maté Organic Energy Shot™ (YM; 140 mg caffeine), or Red Bull Energy Shot™ (RB; 80 mg caffeine)]. Treatments were ingested following a randomized, placebo-controlled crossover design. Participants ran a five kilometer time trial on a treadmill. No differences (*p >* 0.05) in performance were detected with RB (17.55 ± 1.01 min) or YM ingestion (17.86 ± 1.59 min) compared to placebo (17.44 ± 1.25 min). Overall, energy shot ingestion did not improve time-trial running performance in trained runners.

## 1. Introduction

Caffeine (1,3,7-trimethylxanthine) is widely used among athletes, as Desbrow and Leveritt (2007) found that 73% of 140 athletes surveyed at the 2005 Ironman Triathlon World Championships believed caffeine improved performance [1]. Via ingestion of coffee, capsules, or anhydrous powder, caffeine improves performance of moderate to high intensity endurance exercise [2,3,4,5].

However, most studies examining caffeine utilized anhydrous caffeine, which is not readily accessible to coaches and athletes. Additionally, most researchers utilize doses relative to body weight instead of the absolute doses commonly found in commercially available caffeine products. While it could be argued that weighing out a relative dose may be plausible for elite athletes, who have access to dietitians and other trained staff, most track and field/cross country running coaches in the United States have large numbers of athletes to supervise, and this is relatively impractical. Energy drink usage among athletes is also quite widespread, as Woolsey and colleagues reported 48% of 401 collegiate athletes regularly consumed energy drinks [6]. Thus, a coach is far more likely to tell an athlete to “Drink this” prior to a competition instead of calculating a target caffeine dose for each athlete.

Recent research has examined effects of more accessible forms of caffeine, such as energy drinks, on exercise performance in athletes [7,8]. A, yet to be evaluated, caffeine supplement is the energy “shot” which is smaller in volume (generally 59–88 mL), as it lacks the large amounts of sugars, carbohydrates, and/or carbonated water of energy drinks containing caffeine. This low volume and energy content may make their intake practical for runners, who typically avoid supplements due to the onset of gastrointestinal disturbances, as running has a higher occurrence of GI symptoms than cycling [9]. Despite the large amount of literature examining the ergogenic properties of caffeine, few studies have evaluated the efficacy of caffeine ingestion on running performance using ecologically valid assessments, such as time trials.

Therefore, the purpose of this study was to examine effects of energy shot consumption on time-trial performance in trained runners. It was hypothesized that small but meaningful differences in performance would occur with energy shot ingestion because research has shown that low caffeine doses have ergogenic effects during endurance and sprint exercises [2,3,4].

## 2. Methods

### 2.1. Participants

To be eligible for the study, participants needed to be competitive middle distance, and distance runners who participated in cross country, road races, and/or track racing. Nine participants were initially recruited, but three participants withdrew after preliminary testing, due to injury (*n* = 2) or illness (*n* = 1). Thus, six participants completed all testing, and their demographic data are summarized in Table 1. All participants were members of the same post-collegiate running club, and followed similar training schedules each week. The time trials took the place of one of the participants’ weekly workouts. Participants ranged from non-caffeine users (*n* = 1), to moderate consumers of tea and coffee (*n* = 2; 150–200 mg·day^−1^). Participants provided written informed consent, and all procedures were approved by an Institutional Review Board and conformed to the Declaration of Helsinki (CSUSM # 2011-098; CSUC # 2011023).

**Table 1:** Demographic data for subjects (*n* = 6).

Criterion	Mean ± SD (range)
Age (year)	22.5 ± 1.8 (20–25)
VO_2_max (mL·kg^−1^·min^−1^)	69.1 ± 5.7 (60.3–74.4)
% Body Fat	11.4 ± 4.0 (6.6–18.2)
Height (cm)	179.2 ± 7.9 (171.0–189.0)
Weight (kg)	65.4 ± 10.0 (56.0–84.1)
5-km personal best (min)	15.0 ± 0.5 (14.3–15.6)
Weekly training volume in preceding 6 months (km)	94.8 ± 20.0 (65.0–112.0)
Caffeine intake (mg·day^−1^)	80 (0.0, 45, 175, 200.0) *

* Median value + quartiles.

### 2.2. Pretesting Guidelines

Participants were instructed to refrain from caffeine intake a minimum of 36 h pre-visit, which was confirmed through completion of dietary recalls and a brief survey. Participants were also informed to wear the same shoes and not perform intense exercise a minimum of 48 h before visits, treating the time trial as they would an important race or workout session. All experimental testing was performed between 7:00 and 11:00 a.m., with test times being similar among participants (±1 h). Participants were told to follow their normal, pre-race, dietary habits, because it was desired for the time trials to mirror competition as much as possible.

### 2.3. Familiarization Trial

Participants completed four visits during the course of the study. The initial visit included demographic testing (height, weight, and body composition (BC)), a VO_2_max test, and a 3.2-km familiarization trial. Upon arrival, participants completed questionnaires regarding their current training status and training history, health history, and dietary intake, including caffeine intake. Caffeine intake was assessed using a food frequency questionnaire and estimated from manufacturer values and nutritional software (USDA Nutrient Database [10]); however, because the content of coffee beverages varies considerably [11], the habitual caffeine consumption must be interpreted cautiously. Participants arrived after a minimum 3-h fast. Height and weight were measured using a stadiometer and calibrated scale (Health O Meter 402KL Physician Beam Scale; Jarden Consumer Solutions, Boca Raton, FL, USA). Five participants underwent determination of BC via air displacement plethysmography (BodPod^®^, Life Measurements, Inc., Concord, CA, USA). Body fat was calculated with established equations [12]. In the remaining participant, due to relocation of the primary investigator, body composition was determined via the sum of three skinfolds (Lange Calipers; Ann Arbor, MI, USA) at the chest, abdomen, and thigh following standardized procedures [13], and body fat was calculated with standard equations [14].

Subsequently, participants mounted a motorized treadmill (Trackmaster TMX3030C; JAS Fitness Systems/Full Vision, Newton, KS, USA) to warm-up for 3.2 km at a self-selected pace. Speeds chosen for the warm-ups were recorded. After the warm-up, participants were fitted with a heart rate monitor (Polar USA; Lake Success, NY, USA), headgear, mouthpiece (Hans Rudolph 3-way valve; Shawnee, KS, USA), and nose-clips. Indirect calorimetry was used to collect gas exchange data every 15 s during exercise via a metabolic cart (TrueOne 2400; ParvoMedics, Sandy, UT, USA), which was calibrated before exercise according to the manufacturer’s instructions. The VO_2_max test was similar to that described by Smith and colleagues [15]. VO_2_max attainment was confirmed with standardized criteria [16]. After a 5 min cool-down and 10 min of seated rest, participants warmed up for 5 min and then completed a 3.2 km time trial on the treadmill, with the speed starting at 16 km/h and the participants subsequently adjusting the pace as desired.

### 2.4. Treatments

During the next three trials, treatments were randomly assigned in a single-blind, cross-over design using the Latin Squares method. Drinks were 2 ounces (59 mL) in volume, prepared by the primary investigator, administered in opaque containers. Participants were given the option to follow treatment ingestion with a separate bolus of 250 mL of tap water. If the participant chose this for their first trial, it was replicated for all subsequent trials. The placebo (PLA) was an isocaloric “dummy” energy shot flavored to mimic Red Bull™. The other two treatments were Red Bull Energy Shot™ (RB; Santa Monica, CA, USA), containing 80 mg of caffeine, and Guayakí Yerba Maté™ (YM) Organic Energy (Guayakí Yerba Maté, Sebastopol, CA, USA) shot, containing 140 mg of caffeine. Two different energy shots with different caffeine contents were selected to mimic real world scenarios, and this is also why manufacturer’s serving size suggestions (59 mL) were adhered to. Red Bull™ is arguably the most well-known energy beverage, and certainly the most popular (42% of energy drink market in 2009 [17]). However, many athletes tend to consume alternative or organic foods, which is why the YM was chosen as another treatment. YM is all-natural, with its caffeine occurring naturally due to its derivation from the *Ilex paraguarensis* plant, native to South and Central America. The beverage is called *yerba maté* and is widely consumed South America. There are numerous health benefits to YM [18], but no studies have examined potential effects on performance, despite its high amounts of naturally occurring caffeine. Each drink was a different color, but tastes and textures were similar. Ingredients for all treatments at the recommended serving size are demonstrated in Table 2. Subjects were blinded to their treatments, but knew the purpose of the study was to evaluate the effects of energy drinks on distance running performance. Upon completion of testing, participants were debriefed and asked which trial they believed they achieved their best performance and if they could identify the order of their treatments.

**Table 2:** Ingredients of beverages per 59 mL serve. PLA = Placebo; YM = Guayakí Yerba Maté; RB = Red Bull.

Ingredient	RED BULL™ Serving size: 2 ounces (59 mL)	GUAYAKI Yerba Maté™ (Lime Tangerine) Serving size: 2 ounces (59 mL)	PLACEBO 2 ounces Dragonfruit Vitamin Water™, 1 tbsp artificial sweetener, 1 packet Emergen-C citrus-flavored Fizzy Drink mix (4.8 g)
Calories	25	35	20
Carbohydrate	6 g	8 g	4.25 g
Sodium	40 mg	25 mg	60 mg
Vitamin B12	5 μg	100 μg	25.2 μg
Niacin (Vitamin B3)	20 mg	N/A	12 mg
Vitamin B6	5 mg	N/A	0.1 mg
Vitamin B5 (pantothenic acid)	5 mg	N/A	0.5 mg
Vitamin C	N/A	36 mg	1.3 g
Caffeine	80 mg	140 mg	0 mg
Other	Gluconorolactone 1 g Taurine	Proprietary blend: 2.2 g (*yerba mate*) extract, acerola cherry extract, goji berry extract, ginger powder	2.4 mg zinc, 200 mg potassium, 60 mg magnesium, 0.5 mg manganese, 13.2 μg chromium, 98 mg phosphorous, 25 μg vitamin B9, 50 mg calcium

### 2.5. Experimental Trials

Upon arrival, participants completed a 24-h dietary recall sheet to record all food and drink intake prior to the trial. This was photocopied and returned to the participants so that they could ingest the identical items prior to subsequent trials. Dietary patterns between trials were examined using the U.S. Department of Agriculture Nutrient Database [10]. Energy and macronutrient intake did not differ between trials (*p* > 0.05; 3000 ± 236 kcals (12.6 ± 0.99 MJ); 435 ± 38 g CHO, 87 ± 23 g FAT, and 120 ± 30 g PRO). Environmental conditions did also not differ between trials (20.4 ± 0.3 °C; 60 ± 5% relative humidity).

Approximately 50 min after treatment ingestion, participants completed a 10-min warm-up, rested for 5 min, and then initiated a 5-km time trial on a motorized treadmill. We chose ingestion 50 min prior to warm-up so as to provide a total window of ~60 min from ingestion to the start of the time trial, in keeping with common research practices [4,19]. Gas exchange data were recorded continuously (V_E_, VO_2_, VCO_2_, RER) via indirect calorimetry, while heart rate, RPE (0–10 scale, [20]), and treadmill speed were recorded every 3 min. Participants were able to control their speed, but were unaware of their performance time, speed, and distance covered until the last 800 m, when they were able to see the distance remaining.

### 2.6. Statistical Analysis

Data were expressed as mean ± SD, and a two-way repeated-measures ANOVA was used to assess the effects of energy shot intake on variables across time and treatment (SPSS version 17, Chicago, IL, USA). Significance was set at *p <* 0.05. Paired comparisons (Bonferroni) were conducted *post hoc* to identify differences between means if a significant *F* ratio was revealed. Effect size for the *F* ratio was expressed as partial eta-squared (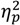). All data are reported as mean ± SD, with minimum-maximum values, or 95% confidence intervals (CI) where appropriate. Additionally, magnitude-based inferences were calculated. This approach was taken because it expresses *p-*values in qualitative and practical terms, and because there are no “truly” zero effects in nature [21]. The smallest worthwhile change calculated was 0.5%. This is midway between the smallest worthwhile change in day-to-day variability in elite runners (0.3 × 1.4% = 0.4; [22]) and the estimated coefficient of variation in our protocol (0.3 × 2% = 0.6; [23]). *P*-values for performance data were calculated using pair-wise comparisons and then entered into a downloadable spreadsheet [24]. Chances were calculated using the following descriptors: <1%, almost uncertainly not; 1%–4%, very unlikely; 5%–24%, unlikely; 25%–74%, possible or unclear; 75%–94%, likely; 95%–99%, very likely; and >99%, almost certainly [24]. These data are reported with their qualitative descriptors in the text, with the percent chances of being beneficial/trivial/harmful in parentheses. Percent changes in means are reported in the text with the numerical change value reported in seconds within parentheses, with 90% confidence limits and Cohen’s standard mean difference (*d*).

## 3. Results

### 3.1. Time Trial Performance

**Figure 1:**
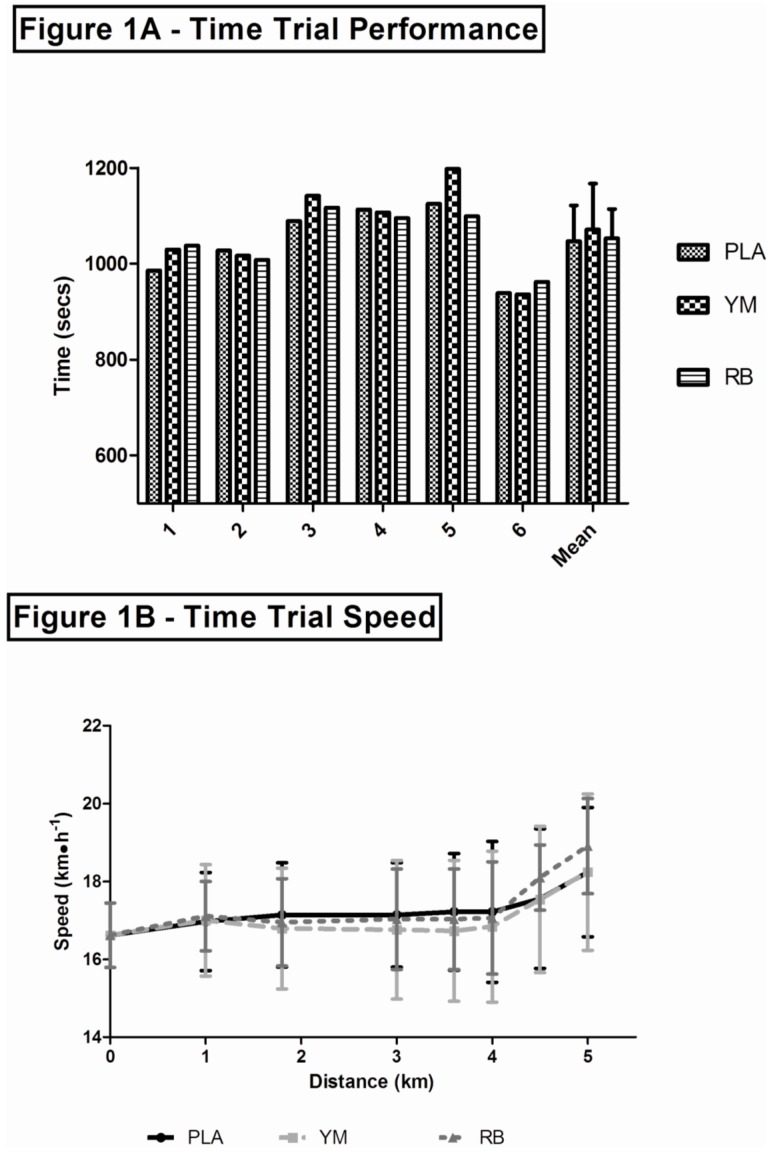
(**A**) Individual five kilometer performance and overall mean ± SD in response to ingestion of RB, YM, or PLA. (**B**) Mean ± SD running speed in response to ingestion of RB, YM, or PLA during TT. PLA = solid black line with squares (■); YM = dotted light grey line with triangles (▲); RB = dashed dark grey line with circles (●).

There was no order effect for the time trials (*F*_2,10_ = 0.09, *η*^2^ = 0.01, *p* = 0.91). Mean ± SD and 95% confidence intervals for performance and physiological variables are shown in Table 3. Individual performances and overall mean ± SD are shown in Figure 1A. Performance was not significantly different between treatments (*F*_2,10_ = 1.44, 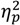 = 0.24, *p* = 0.282). Four participants (66%) revealed the fastest performance in response to caffeine ingestion (RB = 3, YM = 1), while two participants demonstrated superior performance in the PLA trial.

**Table 3:** Mean ± SD and 95% Confidence Intervals for time-trial performance and various physiological variables in response to energy shot ingestion. PLA = Placebo; YM = Guayakí Yerba Maté; RB = Red Bull. TT = time trial; HR = heart rate; RPE = Borg’s Rating of Perceived Exertion.

Variable	PLA	YM	RB
TT Performance (s)	1046.7 ± 74.8 (968.2–1125.1)	1071.7 ± 95.2 (971.8–1171.5)	1053.2 ± 60.8 (989.3–1117.0)
Mean HR (b·min^−1^)	178.4 ± 6.5 (171.5–181.2)	179.2 ± 11.8 (166.8–191.6)	179.4 ± 7.5 (171.5–187.2)
Peak HR (b·min^−1^)	184.3 ± 7.2 (176.7–191.9)	185.0 ± 11.6 (172.8–197.2)	187.5 ± 7.5 (171.5–187.2)
Mean RPE (Borg, 0–10)	6.0 ± 1.1 (4.8–7.2)	5.6 ± 1.0 (4.6–6.7)	6.0 ± 1.3 (4.6–7.4)
Peak RPE (Borg, 0–10)	8.0 ± 2.0 (5.9–10.1)	7.3 ± 1.9 (5.3–9.3)	8.2 ± 1.8 (6.3–10.1)
Peak VO_2_ (mL·kg^−1^·min^−1^)	62.1 ± 6.7 (55.0–69.1)	61.8 ± 7.5 (53.9–69.7)	62.1 ± 7.4 (54.3–69.9)
Peak V_E_ (L·min^−1^)	114.3 ± 21.1 (92.2–136.4)	114.7 ± 25.1 (88.4–141.0)	113.7 ± 16.4 (96.5–130.9)

### 3.2. Physiological Data

**Figure 2:**
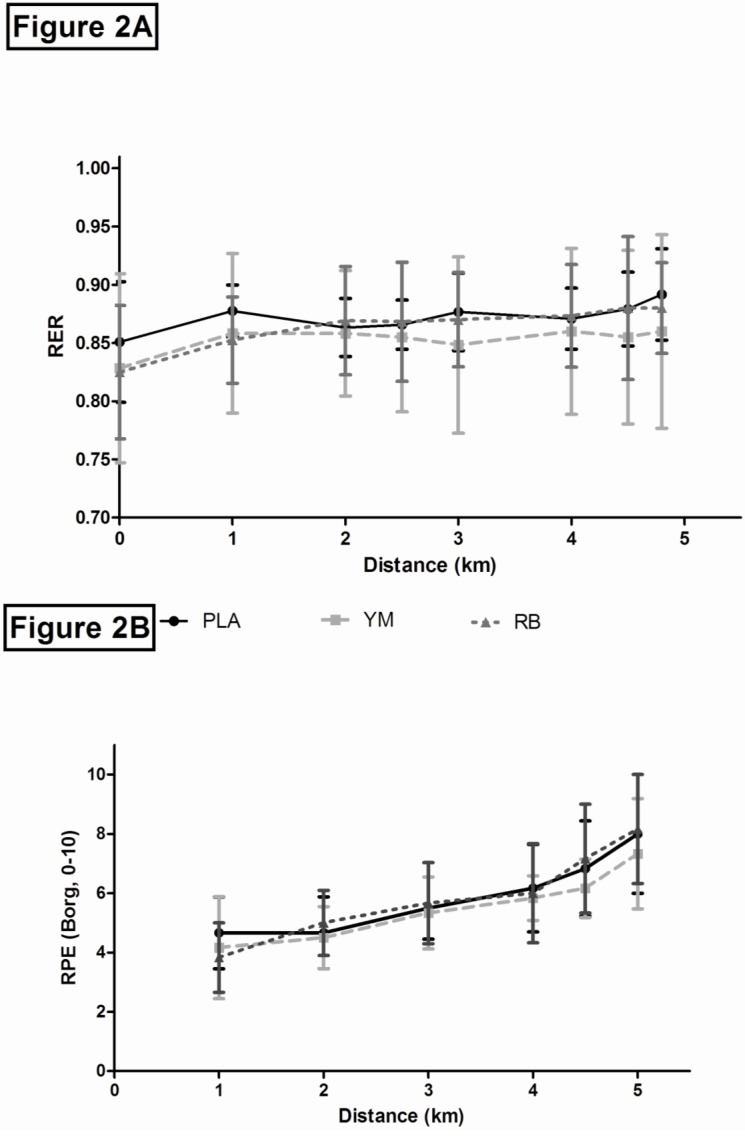
A Respiratory exchange ratio (RER) mean ± SD in response to ingestion of RB, YM, or PLA during TT. PLA = solid black line with squares (■); YM = dotted light grey line with triangles (▲); RB = dashed dark grey line with circles (●). Figure 2B: RPE mean ± SD in response to ingestion of RB, YM, or PLA during TT. PLA = solid black line with squares (■); YM = dotted light grey line with triangles (▲); RB = dashed dark grey line with circles (●).

Running speed increased during exercise (*F*_7,35_ = 4.25, 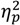 = 0.46; *p <* 0.01) but there was no significant effect of treatment (*F*_2,10_ = 0.925, 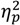 = 0.156; *p* *=* 0.428; Figure 1B). Running speed at one minute prior to the finish, and at the finish, was significantly faster (*p* < 0.005) than the speed at all other data points. *Post hoc* paired *t*-tests showed that the running speed during the PLA trial (17.6 ± 1.8 km·h^−1^) approached significance compared to the YM trial (16.8 ± 1.9 km·h^−1^) one minute prior to the finish (*p* = 0.065). Additionally, at the finish, speed in the RB (18.9 ± 1.2 km·h^−1^) condition also approached significance compared to PLA (18.2 ± 1.7 km·h^−1^) (*p* = 0.093). Oxygen uptake (*F*_7,35_ = 3.77, 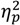 = 0.43; *p <* 0.01), ventilation (*F*_7,35_ = 13.96, 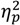 = 0.74; *p <* 0.01), and heart rate (*F*_7,35_ = 99.80, 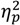 = 0.95; *p <* 0.01) increased across time, but were similar across treatments (*p >* 0.05). Respiratory exchange Ratio (RER) did not differ between treatments (*F*_2,10_ = 0.52, 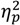 = 0.093; *p =* 0.612) or across time (*F*_7,35_ = 1.602, 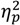 = 0.24; *p* = 0.167; Figure 2A).

### 3.3. Psychological Data

Borg’s Rating of Perceived Exertion (RPE) increased over time during exercise (*F_7_*_,35_ = 15.32, 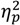 = 0.75; *p <* 0.01), with values at 6, 9, 12, 15 min, and end-exercise significantly higher than 3 min. However, no treatment effects were observed (*F*_2,10_ = 0.76, 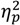 = 0.13; *p =* 0.491; Figure 2B). Post-test responses indicated that blinding was successful, as participants could not distinguish between treatments, although all participants expressed dislike of the PLA and YM solutions. Two participants correctly guessed their fastest trial, but overall, participants could not match trials and treatments.

### 3.4. Magnitude-Based Inferences on Performance Data

Despite no significant differences in performance, magnitude-based inferences revealed that the PLA condition was likely to provide a small performance improvement (% chance Beneficial/Trivial/Harmful: 92/8/0%) compared to YM, as the mean for the PLA condition was 2.3% (25.0 ± 29.6 s; Cohen’s *d* = 0.30) faster than YM. Comparisons between the PLA and RB were unclear (65/30/5%), with these treatments differing by only 0.7% (7.0 ± 26.4 s; Cohen’s *d* = 0.10). However, the RB condition also showed a small, likely improvement compared to YM (82/16/2%), with mean RB performance 1.7% (18.5 ± 35.6 s; Cohen’s *d* = 0.24) faster than YM.

## 4. Discussion

The primary purpose of the present investigation was to examine the effects of caffeinated “energy shots” on the distance running performance in trained runners. To our knowledge, this is the first study to evaluate the effects of energy shot consumption on exercise performance. Results showed no change in five kilometer, time trial performance between treatments. Mean performance for the PLA trial was 2.3% faster than the YM trial and 0.7% faster than the RB trial, while performance with RB was 1.7% faster than in the YM trial. There was no significant effect of energy shots on speed, HR, RPE, VO_2_, or V_E_ compared to placebo.

The dose of caffeine administered in this study (1.43–2.5 mg·kg^−1^ BM for the lightest participant and 0.95–1.66 mg·kg^−1^ BM for the heaviest participant) is below what may be a threshold dose (>2.5 mg·kg^−1^ BM) for caffeine to elicit an ergogenic response [25]. Our data showing no significant effect of caffeine-containing energy shots oppose results of Bridge and Jones (2006) and O’Rourke and colleagues (2008), who found that ingestion of anhydrous caffeine mediated improvements in performance in eight kilometer and five kilometer time trials, conducted in a field setting [5,26]. The ergogenic effect reported by these authors represents a 4 s·mile^−1^ (2.5 s·km^−1^) faster pace, which would benefit any runner [5,26]. Our dose was lower than the 3 and 5 mg·kg^−1^ doses used in previous studies, which may have contributed to the lack of ergogenic effect [5,26]. However, previous results show that low doses ranging from 1.3 mg·kg^−1^ to 3 mg·kg^−1^ improve performance by 1.3%–3.0% during prolonged cycling and sprint cycling [3,4,27]. Vandenbogaerde and Hopkins (2010) recently reported that 100 mg of CAFF (~1.3 mg·kg^−1^ BW) improved swimming performance in elite swimmers by 1.3% [2]. These data show that the belief in a caffeine “threshold” may be a bit premature; and thus, more work should examine lower caffeine doses on exercise performance, since lower doses will more accurately reflect amounts of caffeine consumed by athletes (*i.e.*, in energy drinks or cola beverages).

Previous studies examining the efficacy of energy drinks for improving exercise performance have yielded equivocal results. A recent study demonstrated that 500 mL of RB (160 mg CAFF, 2 g taurine) improved cycling performance [7]. However, the effects of energy drinks on high-intensity exercise and sprint performance are unknown. For example, Astorino and colleagues reported that ingestion of one serving of RB (250 mL containing 80 mg caffeine) had no effect on repeated sprint performance in female collegiate soccer players [8].

The purpose of this study was not to examine the mechanisms of caffeine action, but mechanisms such as adenosine antagonism, reduced perceptions of exertion and pain, enhanced motor unit recruitment, and greater preservation of strength, have been postulated as ways that caffeine exerts its ergogenic effects [28,29,30,31,32].

One limitation of the present study is the small sample size. The study population was a convenience sample recruited from a group of elite local runners who had to meet minimum performance and training criteria to be eligible for participation. A follow-up study with more participants is needed to confirm these findings before recommendations on energy shot usage can be made to coaches and athletes. Another limitation was the decision to perform the time trials on a treadmill. While steady-state running can be adequately simulated on a treadmill, the demands of a time trial are more difficult to meet. Nevertheless, previous studies are characterized by treadmill time trials, as Wiles and colleagues demonstrated, improved (1.5%) 1500-m performance after ingestion of 2.5 mg·kg^−1^ caffeine [33]. Furthermore, despite efforts to familiarize athletes with the treadmill, it is possible they were still uncomfortable in this setting; this may have prevented them from achieving a truly maximal effort, as their performance times in the lab were considerably slower than their personal bests. However, feedback from the participants after data collection indicated that despite the longer time needed to cover five kilometers on a treadmill, the time trial adequately simulated (at least perceptually and psychologically) the challenge of a five kilometer track race.

Finally, the coefficient of variation for performance (2.1%) must be considered. We calculated this value by extrapolating the time of the 3.2 km familiarization trial to a full five kilometers and compared this time with the PLA trial. This value is similar to what has been previously reported for five kilometer treadmill time trials in well-trained runners (2.0%; [23]). However, performance differences did not reach the magnitude for the estimated smallest worthwhile change when the CV was considered.

## 5. Conclusions

Ingestion of two commercially available energy shots with differing levels of caffeine does not alter treadmill five kilometer time-trial performance, RPE, or physiological variables in well-trained runners, compared to a placebo. The results must be considered preliminary due to the small sample size, and further studies should examine the effects of energy shot ingestion on performance in a variety of sport and field settings. While some research has been conducted on energy drinks, this is the first study to examine the efficacy of energy shots on exercise performance. Therefore, additional study is needed to examine their long-term effects on body composition and health, in addition to athletic performance, before recommendations about their usage can be provided to coaches and athletes.

## Declaration

Abstracts of these data were presented at the annual meeting of the Southwest Chapter of the American College of Sports Medicine, November 2011, and the ACSM annual meeting, June 2012.
